# Meeting of the Fulton County Medical Society

**Published:** 1915-03

**Authors:** R. R. Daly


### FULTON COUNTY MEDICAL SOCIETY.

### Regular Meeting March 4, 1915.

By R. R. Daly, M. D.

Dr. L. M. Gaines showed a case of Specific Myelitis of the Cord. He demonstrated the absence of power and the changes in the reflexes as they have clinically appeared and gave a history of the case with the difficulty of getting a diagnosis. A specific infection was admitted by the patient readily enough, but it had to be fitted to the conditions in a real etiologic relation and this was well done.

Dr. Hansel Crenshaw said that it is an interesting question in these cases as to why in the early stages there are loss of reflexes and flaccid paralysis, while later on there are spasticity and exaggerated reflexes. The answer is that at first the inhibition by the upper neurons is excited as if to protect the parts in disease, while later this relation is destroyed by the progress of the lesion and the uncontrolled lower portion of the cord dominates the condition. Bevor's sign was not mentioned. It is the drawing up of the umbilicus because the rectus muscle is not affected above, but is paretic below that point. When Gaines made the distinction between medullary and meningeal inflammation he was correct in saying that in the medullary type, there was no lymphocytosis.

Dr. J. M. Brawner asked if X-Ray showed any disturbance in the vertebral column and also if salvarsanized serum had been used. One of his own cases of Syphilitic Plastic Paraplegia had been given salvarsan intravenously and had not improved. Later he did well with the serum. It has been remarkable to note the selective action that the virus has in the cord. The pyramidal tract is chosen very often, while the direct cerebellar tract is selected only occasionally. Another selection occurs in Locomotor Ataxia.

Gaines said that he did not use Bevor's sign because of the extreme weakness of the patient. The X-Ray shows prominent vertebra at the seat of the pain, but nothing more. Salvarsanized serum is still an open question with him. He has not always been satisfied with it, but he is sure that neosalvarsan in the cord is not safe. The injection would probably do no good in this case because of the thrombotic softening and destruction that has taken place.

The differential diagnosis of tumor of the cord in this case was difficult. Bladder troubles came early but were not symmetrical with the affection of the muscles. Ataxic Paraplegia also was set aside because there was no early spacticity. We have the well defined zone of hyperasthesia, the location of the pain in the back and the fact that there is syphilis. These together with the other symptoms, point to the diagnosis already made, but there remains the possibility of diffuse gumma.

Dr. I. T. Catron and Dr. John Wallace reported a case of Tumor of the Anterior Crural Nerve. The tumor was removed and shown. The young man had given a history of a swelling in the groin a year before, which went down with poulticing. In January, it returned and looked like a bubo. There was no specific history. Later it became painful and operation was done to determine if there were any hernia. The anterior crural nerve was four times its normal size and showed the tumor exhibited. This was removed successfully and the patient now walks with a slight limp.

Dr. Allan H. Bunce said he had made a microscopic examination of the tumor. The whole thing looked like a cystic inguinal gland, but sections showed conclusively that it was a neuro blastoma coming from the nerve cell. There are almost as many kinds of nerve tumors as there are variey of cells in the nerve as a whole. The exones and dendrites, the neurones, the neuroglia, the lining cells of the sheath of Schwann may all have tumors and they can be differentiated if pressure has not been too great upon this characteristic cells. The tumor exhibited is rare and is seen only in the adult.

Dr. W. S. Goldsmith exhibited a case of Tetanus treated with serum, well in 19 days and discharged in 46 days. He was also given bromide and chloral as indicated. He had 58.000 units of antitoxin costing $90. The history is this:

On January 1, 1915, an overgrown, lanky negro boy, 15

years old, a driver of a coal wagon, was brought to the hospital complaining of inability to open his mouth wide. Shortly after his admission he had a convulsion lasting about five minutes. During this convulsion his muscles were in a state of tonic contraction and there was slight opisthotones.

Examination revealed a small wound on one finger and a slight contusion of his right great toe. These wounds were caused by a piece of coal.

A diagnosis of tetanus was made and patient was given 5,000 units tetanus antitoxin hypodermically.

Upon admission patient's temperature was 101, pulse 122, respiration 24.

He was given sodium bromide gr. 20 with chloral hydrate gr. 10 by mouth and sodium bromide gr. 40 with chloral hy-hydrate gr. 20 per rectum, every four hours. In addition to this he was given morphia gr. 1-6 per hypo. every four hours. The next day at 4 p. m. his rectal temperature was 105, pulse 136, respiration 24. He received 12,000 units antitoxin intraspinously. Phenacetin gr. 5, also given. At 8 p. m. his temperature was down to 103.

Patient became semi-comatose and his temperature ranged from 101 to 103 for two days, when 6,000 units antitoxin again given intraspinously at 4 p. m. At 7 p. m. his rectal temperature was 106 4-5, pulse 130, respiration 24 and patient was talking at random. Opisthotonos rather pronounced. Patient now averaging two to three convulsions daily. His temperature came down to 104 under influence of phenacetine and stayed at 104 all night. Next day 6,000 more units given intraspinously, followed by a slight rise in temperature.

The next day the opisthotonos was so pronounced that patient had to be placed under the influence of chloroform before the spinal canal could be entered. At this time he was given 6,500 units. On this day his temperature was down to 102 3-5. Temperature again went to 104 2-5 following an injection of 6,500 units the next day. He received 6,500 units every day for the next four days, but temperature did not rise above 103 following any of the injections. Patient continued very restless and with opisthotonos present until January 10, when his temperature came down to 99 and he had a quiet night's sleep. However, the next day his nervous symptoms again appeared and his temperature ascended to 104. Four days later he became quiet and remained so during the remainder of his stay in the hispital. The bromides and chloral were gradually reduced and finally omitted about the 15th day, an occasional dose of morphia being given when indicated. On the 17th day he was given soft diet and nutritive enemata began. On the 19th day his temperature reached normal and stayed there. He improved rapidly and on the 24th day was put on full diet with forced feeding. On the 30th day he got in a roller chair and 6 days later was able to walk unassisted. He was discharged, cured, on the 46th day. Total antitoxin 58,000 units.

Laboratory examinations:

Urine—Negative.

Spinal fluid—Negative.

Wasserman's—Negative (blood and fluid.)

Blood count—Normal.

Dr. Goldsmith also showed a case of Aneurism of the Descending Aorta with unusually strong pulsations felt through the back.

Dr. E. L. Griffin said that he had had a case of Tetanus get well in 10 days treated with injections of 60 minims saturated solution magnesium sulphate repeated many times.

Dr. W. P. Nicolson asked if there were really any evidence that the serum helped this case. In the early part of his practice he had cured two cases of tetanus with just the chloral and bromide. He knows that the antitoxin is prophylactic and gives it every credit for that action, but he is far from being convinced that it is of value in treatment.

Dr. E. C. Cartilege said that he had a case cured by carbolic acid.

Dr. L. S. Hardin said that after the tetanus has reached the brain, he can not understand how it can be cured or the infection destroyed.

Goldsmith said that the best evidence of the cure was the patient who was present. As to how the antitoxin cures or works, we do not know, but it does have its effect in prevention and he is convinced that it is of value in treatment.

Dr. C. C. Aven exhibited a case of Multiple Arthritis in a man of 44. His history was unimportant except that he had had some "rheumatism" and one attack of gonorrhoea. In July he began suffering pains in his various joints and had great difficulty in breathing. He was treated for this and had to go to Grady Hospital. A diagnosis of infection from Riggs Disease was made and he improved after treatment of his teeth, so that he could leave the hospital, but he was far from well and the pains returned. He went to college and it was found that Wasserman was 4 plus though he did not admit luetic history. He was treated with several injections of venarsen and has improved steadily. The injections were given at four-day intervals six times.

Dr. L. M. Gaines said that the man might well have had syphilis without knowing it because a chancre is not always present. Moreover, the chancre sometimes is intra urethral. Inherited syphilis may show up in such cases in the late thirties.

Dr. Cosby Swanson said that about one in five cases of syphilis is infected through the mouth and the victim doesn't know it. He has used venarsen in four cases without good results.

Dr. J. N. Brawner asked if anyone knows the composition of venarsen.

Dr. A. H. Bunce said that the fact that the case went so long without discovery of the lues illustrate the danger in not making Wasserman a routine matter.

Aven said in closing that a positive Wasserman means syphilis usually and he would like to have a routine in all his cases. He doesn't care to discuss venarsen farther than to say he has been using it in 100 doses at the college and there have been no bad results. He knows nothing more of its composition than the label tells. He exhibited another case showing Pleurisy with effusion very low in the back. It had been diagnosed as Lumbago, but aspiration relieved it completely.

Dr. W. P. Nicolson talked on the Surgery of the gall bladder and showed several cases. Among them was a man who had suffered severe toxic neuralgia of the knee that was thought at one time to be a floating body there. The appendix was tender and at the operation for its removal the gall bladder was drained. The patient improved markedly, but suffered a relapse in about a year. The bladder was then removed and recovery was complete. The patient was shown.

Another case was an old personal friend who had constant headaches. They disturbed his whole life and caused him to wear a frown. He traveled around the world and gave up business because of the pains. Many laboratory tests were made and consultations held. It was found that he had myocarditis of the auricle but that did not assist any. Eventually he was operated upon because of his toxicity and the appendix and gall bladder removed. He got well quickly and has been well and free from pain for several years. Whenever one has a toxic condition to deal with, the source must be found. If search is painstaking and careful, it will be rewarded. Chronic inflammation of the gall bladder is very common in man and it has several classic symptoms. Pain over the gall bladder, pain in the right shoulder, muddy complexion and very likely some irritation around the appendix all point to cystitis, but it is not always that the classic symptoms appear. The gall bladder must be kept in mind and studied in every case of indigestion and toxicity.

Dr. E. L. Griffin said that the early discovery and removal of diseased conditions of the appendix and gall bladder are imperative. The wide extent of the injury due to toxic states arising in the gall bladder is remarkable and it should never be out of our mind. Diseases of the joints, eye, throat, nerves and many other organs remote from seeming relation to the gall bladder were menaced by cystitis and their only protection was in our recollection that they might be disturbed in this way.

Dr. E. D. Highsmith said that it is well to remember that while an appendix can never do any good and no harm can come from its removal, the gall bladder does have a function. We should be conservative as to its removal and thoughtful as to its surgery. He did not decry operating there to remove dangerous conditions, but advocated conservatism.

Dr. L. P. Daly agreed with Highsmith as to the surgery of the gall bladder. In empyema it is necessary to remove it and do so promptly, but he does not believe that even drainage is a good routine measure in connection with appendectomy.

Dr. C. W. Strickler said he had seen both of Nicolson's cases and there was no doubt as to the improvement related. The removal of the gall bladder was necessary in each instance. In the case with myocarditis, the heart disease was there surely enough, but it probably was due to the toxicity of the gall bladder. The removal of the appendix is not enough when the gall bladder is affected.

Dr. M. T. Davis told of a case who had come to him stating that she had passed 200 gall stones since January. She had been taking some patent medicine and was mistaking the cholesterine coagulums for gall stones.

Dr. Sage Hardin told of the difference of opinion upon this matter at the Mayo's clinic. One was operating and the other declining to do so.

Dr. C. E. Boynton said that his own case was peculiar. He doubtless had cholecystitis and twice he prepared for operation, but each time something unforeseen prevented the work. He went to the mountains and recovered.

Dr. J. C. White said that he personally had 15 years of gall bladder trouble. He was drained and got better, but in 1909, he had the cyst removed and has been well since then.

Nicolson said in closing that no one who holds a gall bladder in his hands can tell what is the condition inside. Cholecystectomy is not to be advocated in all cases by any means. It is a big operation and must be approached with care. The wider one's experience is the better he can judge of this work. He is satisfied that to cure gastric ulcer, the gall bladder must be drained and the appendix removed. He drains them for about twelve days. They close all too quickly as a rule. He doesn't believe in "frills" in the technic. He ties the stump around the tube and drops it back into the abdomen.

Dr. L. Sage Hardin related a case of Cirrhosis of the Liver with many classic and a few peculiar symptoms. The history was alcoholic and luetic. He operated and could do nothing more than to curette the superior surface of the liver and fasten it to omentum. It was very hard indeed, and one could put a needle through only with difficulty. The result was astonishing in that there was great improvement of the patient and alleviation of the symptoms. The man is around town today.

Dr. C. E. Boynton described in a preliminary report three cases of Congenital Pyloric Stenosis. He had never seen any cases before and was at a loss what to do with them. He displayed several minute gold seeds or ovules attached as guides to very slender tubes by means of which he was able to pass through the stenosis and keep the children alive. The mechanicle devices were very ingenious and they were displayed at the meeting.

Dr. G. M. Niles said he wanted Boynton to present the case because it was unique and covered in a unique way. Among other things the apparatus was seen to go into the pylorus by means of the fluoroscope. Southern men have done as many fine things and as many ingenious and new things in medicine and surgery as any set of men in the world, but it happens that less has been said about them. He wants these cases recorded as having been done by sight.

Dr. Howard Bucknell said he has seen the pathology of several such cases. Atresia is present in some and invagination in others. Real stenosis is a spastic condition. Pseudo valve formation occurs as the membrane gets loose and invaginates. Still's statistics in King's College, London, are that one-half the gastric cases die. In the others a part have gastro enterostomy and another group has the pylorus stretched in some way.

### REGULAR MEETING MARCH 18, 1915.

The program was occupied by the guest of the evening, Dr. C. C. Bass, of New Orleans. His talk comprised three subjects: The Bass-Watkins Typhoid Agglutination Test, Fundamental Facts of Importance in the Life of the Malaria Plasmodia bearing upon the Treatment of Malaria, and The Cause and Treatment of Alveolodental Pyorrhea.

His explanation of the typhoid test was that it could be used at the bedside without a microscope if properly modified from the Widal method. His special improvement upon the Widal test is in bringing the bacilli of the test solution so closely together that the agglutination will take place not only quickly but in so great a degree that the clumps may be seen on the slide with the naked eye. In other words he crowds into the test solution a thousand or more times as many bacilli as are used in the Widal and thus because of their greater proximity to each other the mutual attraction or affinity they have for each other under the conditions, has less space resistance to overcome and the clumping occurs promptly and in great degree. He demonstrated the test before the Society.

In talking of Malaria, he showed the developmental cycle of the Plasmodia and stated that it was the belief of himself and fellow workers that it really entered the red cells and grew within them rather than on the surface. His special point, however, was made in answering the question as to what became of the plasmodia when they seemed to disappear from the blood stream as in the estivo-autumnal type yet continued to produce their effects. He first showed how in the capillaries the walls are indented by the nucleii of the cells making the walls and the lumen made very irregular. This is natural. The blood can flow through these restrictions readily and the normal red cells have no trouble in passing because they are soft and nearly as fluid as the serum itself. The malarial parasite, however, has much greater resistance and the red cell when filled with them and nearly ready for their segmentation, is harder and can not pass the restriction in the capillaries. The vessel is plugged for the time and if it happened to be in large numbers in the brain where the circulation is terminal, there would be anaemia and possibly coma resulting. In whatever tissue this occurs, the life cycle of the parasite continues and different results follow the ultimate rupture of the invaded red cell according as to whether the break occurs upward where there are other red cells to be reached by the wandering germ, or whether the chief opening is downward past the construction where there is serum rendered devoid of red cells by the plugging. In the former case the germ can live because its quickly meets the red cell necessary to its sustenance. In the latter, it will die because of the destructive action of the plasma. The importance of all this regarding the treatment depends upon the fact that plasmodia are for a time isolated from the blood current saturated with quinine and thus they may escape. Repeated treatment is needed until there can no longer be a recess in the whole circulation where the quinine has not gone. In this way only can the plasmodia all be killed.

The subject of Pyorrhea made the crux of the evening. The Dental Society had been invited and a large number of its members were present. It would be futile to attempt to epitomize what Dr. Bass said on this very important subject and the proofs he gave that his thesis was true. He was convincing. His various publications will tell the story clearly. Let it be said that he stated without qualification that the endamoeba buccalis was the cause of Pyorrhea alveolaris and had pyorrhea unless the endamoeba were there. Moreover,, that whenever the endamoeba were present in the mouth the host had pyorrhea whether he knew it or not and that he never 95% of adults have pyorrhea in some degree. Many of them know it, but more do not.

He demonstrated by charts and illustrations how this could be and he showed the method of invasion and the development of the disease. The particular and striking teaching is that the ameoba act as the vehicles by means of which the tissues are invaded by pus-producing micro-organisms. Thus, at the bottom of every pocket in the peridental membrane, no matter how small it may be, there is a small ulcer. That is there is granulation tissue and loops of capillaries. This tissue contains certain brown stained bodies the nature of which is not clearly known, which comprise the food of the amoeba. The amoeba is motile and very much alive. It burrows all through the granulation tissue and beyond it into the healthy tissue in search of its food. Now the endamoeba is considerably larger than the bacilli that produce pus and it seems to have the faculty of making these bacilli cling to it. So when it leaves the pus pocket above the ulcer and begins to burrow for its food in the deeper tissues, the pus organism is carried into new fields where it otherwise could not go, and the disease is just that much farther extended.

These amoeba are so deeply seated in pus pockets of any size that it is practically impossible to eradicate them. The pockets should be treated surgically and all over hang of dead alveolus as well as scale on the teeth must be removed, but even then there is likely to remain many of the amoeba. They can be destroyed by strong acids, powerful solutions of iodine and other destructive agents that are dangerous to the patient. They can also be promptly killed by emetine when this is injected either hypodermically or intravenously. This does not disturb the patient or imperil him in any way. In pockets that are so small as to escape the expert observation of the dentist, the disease is the same as in the larger pockets except in degree. These can not be treated surgically, because they are not discoverable. We know they are present only because of the history of the disease, which is one of gradual growth from sources and at foci unknown until the prominent symptoms announce it. These small foci can be cured and all the amoeba contained in them killed by emetine and in no other way at present known.

Prophylaxsis should always be practiced and so far as possible the children should be protected from infection from their elders. Just how best to do this is a big question and one not readily answered. It will depend ultimately upon education of every one as to the great prevalence and the immense damage done by this disease. Once people know the dangers both to the jaws and to the whole system coming from the poisons absorbed, they will take almost any measure to prevent it. Meantime, clean teeth, prompt treatment of pyorrhea and great care in conveying infection from one to another by contact and food utensils are recommended. The essential thing is recognition of the cause of the disease and its ultimate treatment by emetine.

